# Parallel vector memories in the brain of a bee as foundation for flexible navigation

**DOI:** 10.1073/pnas.2402509121

**Published:** 2024-07-15

**Authors:** Rickesh N. Patel, Natalie S. Roberts, Julian Kempenaers, Ana Zadel, Stanley Heinze

**Affiliations:** ^a^Lund Vision Group, Department of Biology, Lund University, Lund 22362, Sweden; ^b^Nano Lund, Centre for Nanoscience, Lund University, Lund 22362, Sweden

**Keywords:** navigation, insect, path integration, memory, bumblebee

## Abstract

Insects rely on path integration (vector-based navigation) and landmark guidance to perform sophisticated navigational feats, rivaling those seen in mammals. Bees in particular exhibit complex navigation behaviors including creating optimal routes and novel shortcuts between locations, an ability historically indicative of the presence of a cognitive map. A mammalian cognitive map has been widely accepted. However, in insects, the existence of a centralized cognitive map is highly contentious. Using a controlled laboratory assay that condenses foraging behaviors to short distances in walking bumblebees, we reveal that vectors learned during path integration can be transferred to long-term memory, that multiple such vectors can be stored in parallel, and that these vectors can be recalled at a familiar location and used for homeward navigation. These findings demonstrate that bees meet the two fundamental requirements of a vector-based analog of a decentralized cognitive map: Home vectors need to be stored in long-term memory and need to be recalled from remembered locations. Thus, our data demonstrate that bees possess the foundational elements for a vector-based map. By utilizing this relatively simple strategy for spatial organization, insects may achieve high-level navigation behaviors seen in vertebrates with the limited number of neurons in their brains, circumventing the computational requirements associated with the cognitive maps of mammals.

## Significance

Insects navigate by utilizing path integration (vector-based navigation) and landmark guidance for homing. Bees also exhibit more complex behaviors, including creating novel shortcuts between locations. Though novel shortcuts have historically been indicative of a cognitive map, its existence in insects is highly contentious. Investigating condensed foraging behaviors in walking bumblebees, we reveal that multiple vectors learned during path integration can be transferred to parallel long-term memories and that these vectors can be recalled at a familiar location for homeward navigation—both fundamental requirements of a vector-based mental map. Our demonstration of the flexible use of long-term navigation vectors provides a plausible foundation for how bees achieve vertebrate-like, high-level navigation behaviors with far fewer neurons in their brains.

Insects are impressive navigators, rivaling the abilities of mammals. Bees and ants in particular can home in direct paths after extended convoluted travel (1, 2). These insects largely rely on a neural computation termed path integration to continually compute a vectorial representation of the most direct path back to the origin of its excursion in working memory (3), an ability shared with many other animals (1, 2, 4–9). While path integration is a powerful tool for directed navigation, bees perform navigational feats that extend past homing—they have been shown to make novel shortcuts between locations (10) and construct optimal routes between multiple places, i.e., “traplining” (11–13). The ability to construct novel shortcuts has often been regarded as evidence for the presence of a “cognitive map,” a single spatial representation of an animal’s surroundings (14–16).

A centralized cognitive map is widely accepted in mammals (17, 18). However, its existence in insects is highly controversial (15, 16, 19–22). Critics have instead hypothesized that insects are able to perform behaviors indicative of a cognitive map by pairing visual information from familiar locations with path integration-derived vectors and storing this paired information in long-term memory. In this model, when an insect wishes to travel to a food location from the nest, an associated food vector is retrieved and can be used for navigation to the food site. Further, if an animal wishes to travel between two known locations, it can add a memorized food vector associated with one location to the state of the path integrator at the other location, thereby creating a novel shortcut (23–25). We extend this idea by suggesting that if home vectors are stored and associated with familiar locations, an associated home vector can be retrieved once a familiar location is encountered and can be used for homeward navigation. Further, if an animal wishes to travel between two known locations, it can add memorized home vectors from both locations to construct a new vector to guide it between places. In doing so, the animal would have a flexible decentralized mechanism with which to construct a map composed of independent vectors anchored to the world by nodes of familiar locations. This would allow for complex navigation behaviors without the need for a centralized, classical cognitive map. We term this idea a “vector map.”

Navigational models reliant on vector computations can successfully replicate hymenopteran behavior (25–27). Further, when adding hypothetical neurons capable of storing long-term vector information to a neuroanatomically grounded model for path integration (28), novel shortcut building and traplining can be recapitulated (29). Despite their gains, these models are reliant on assumptions that are not yet supported by behavioral evidence. First, it is difficult to know which previous routes a foraging insect has experienced in nature. Therefore, many complex behaviors bees exhibit in their natural habitat, including the recollection of potential vector memories (30), may simply be the results of routes reliant on landmark guidance (21, 31). Second, though behavioral evidence of food vector recollection for directed navigation exists (1, 32–34), clear evidence of long-term storage and recall of home-directed vectors from a goal learned during path integration is lacking, making the idea of a vector map completely theoretical (35).

Using a behavioral assay that condenses foraging behaviors in bumblebees walking in a controlled laboratory-based setting (9), we reveal that hive-directed vectors learned during path integration can be transferred to long-term memory, that multiple such vectors can be stored in parallel, and that these vectors can be recalled at a familiar location. These findings demonstrate that bees possess the fundamental capabilities required for constructing a vector-based map of their environment.

## Results

### Bumblebees Orient Unpredictably when Foraging under an Unfamiliar Pattern of Polarized Light.

**Fig. 1.**
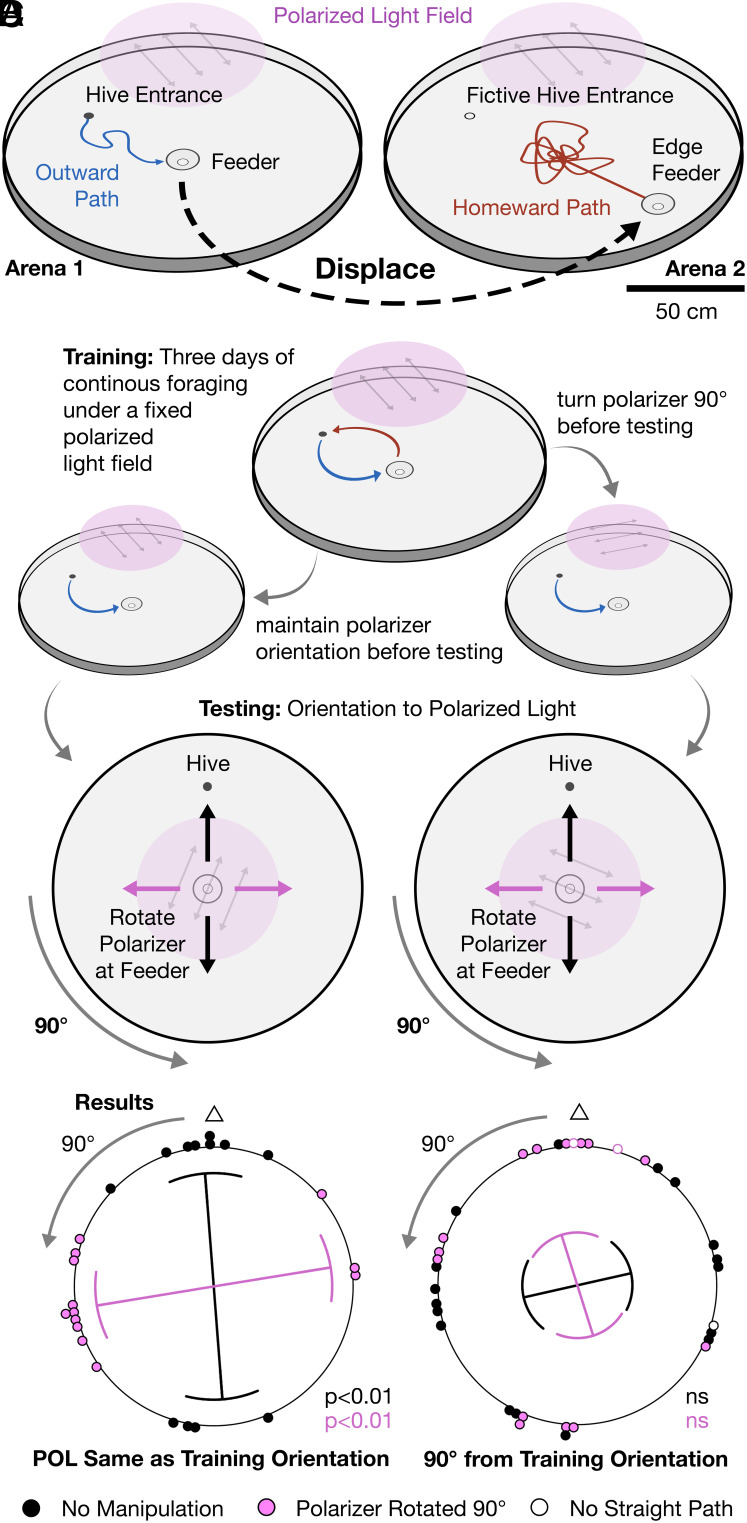
Bumblebees orient unpredictably when foraging under an unfamiliar pattern of polarized light. (*A*) A circular arena (150 cm diameter) was connected to a hive via an entrance located 25 cm from the arena’s periphery. Sugar water and pollen were provided in a conical feeder at the arena center. The feeder and hive entrance were connected to the arena below ground level. An overhead polarized light source provided illumination. Schematic tracks show results from a previous study that demonstrate that bees use path integration to generate home vectors of defined directions and lengths (9). (*B*) Current experimental design: Polarizer was either kept in place or offset by 90° following 3 d of training. Bees then located and were trapped at the central feeder. The polarizer was then kept in the same position or rotated by 90°. Integrated paths should result in identical home vector orientations for both initial polarizer positions. (*C*) Homing bees exhibited significant orientations when the polarizer was initially oriented in the same position during both training and testing days [bimodal orientations are expected from a polarization stimulus, thus data were doubled to create unimodal distributions; fixed: 348.05 ± 40.2°, *P* < 0.001; rotated: 161.75 ± 32.6°, *P* < 0.001; data redisplayed from Patel et al. (9)]. (*D*) Significant orientations were no longer observed when the polarizer was offset 90° between training and testing days (doubled data; fixed: 150.115 ± 78.09°, *P* = 0.069; rotated: 349.29 ± 79.87°, *P* = 0.135). Lines within each plot: circular means; curved sectors: circular SD; line lengths: strength of orientation (R-bar); triangles above plots: hive direction.

Previous experiments revealed that bumblebees perform vector navigation while walking over short distances, constructing home vectors of defined lengths and orientations by path integration, and that these vectors are stable for at least 12 h (9). During those experiments, bumblebees reliably used polarized light to orient in a featureless, symmetrical arena (Fig. 1*A* and *SI Appendix*, Figs. S1 and S2) when tested via the following procedure: An overhead polarized light field was fixed in place during three-days of training, during which the bees could freely forage. During subsequent testing, the polarizer was initially positioned as during training and individual bees were allowed to enter the arena and locate a symmetrical, conical feeder set below the plane of the arena floor at the center of the arena. Importantly, the symmetry of the arena and the feeder results in a lack of visual panoramic cues from the center of the setup (*SI Appendix*, Fig. S2). Once at the feeder, bees were trapped and the polarizer was then either kept in the same position or rotated by 90° (Fig. 1*B*). When the polarizer was not rotated, bees would correctly orient homeward paths in the hive–feeder axis. When the polarizer was rotated by 90°, homeward paths were oriented perpendicular to the direction of the hive (Fig. 1*C* and *SI Appendix*, Table S1). Further, when the overhead polarized field was replaced with a diffuse one lacking polarization information, home vectors were no longer observed (9).

Using these previous results as our foundation, we now asked whether the orientation of homing paths was determined by the bees’ foraging experiences during the three-days training period or by stimuli experienced during the outbound journey of the test trial, as expected for path integration. To do so, we replicated our previous experiment, but now also rotated the polarized field 90° relative to its training orientation before bees left the hive during testing trials. If bees solely oriented according to path integration-based home vectors built up in working memory during their outbound trips, we predicted results equivalent to those from our previous experiments. Surprisingly, we found that when the polarizer was offset by 90° between training and testing days, significant orientations of homeward paths were no longer observed—in fact, bees tended to orient opposite of our predicted directions (Fig. 1*D* and *SI Appendix*, Table S1). Bees thus oriented predictably to a polarized light field only when the angle of polarization aligned with the polarization field experienced during the previous days of training. This suggests that the bees are guided by a combination of information from the training period and their home vector, leading to homing failure if the two conflict.

### Path Integration Working Memory Is Not Required for Homing in a Featureless Environment.

**Fig. 2.**
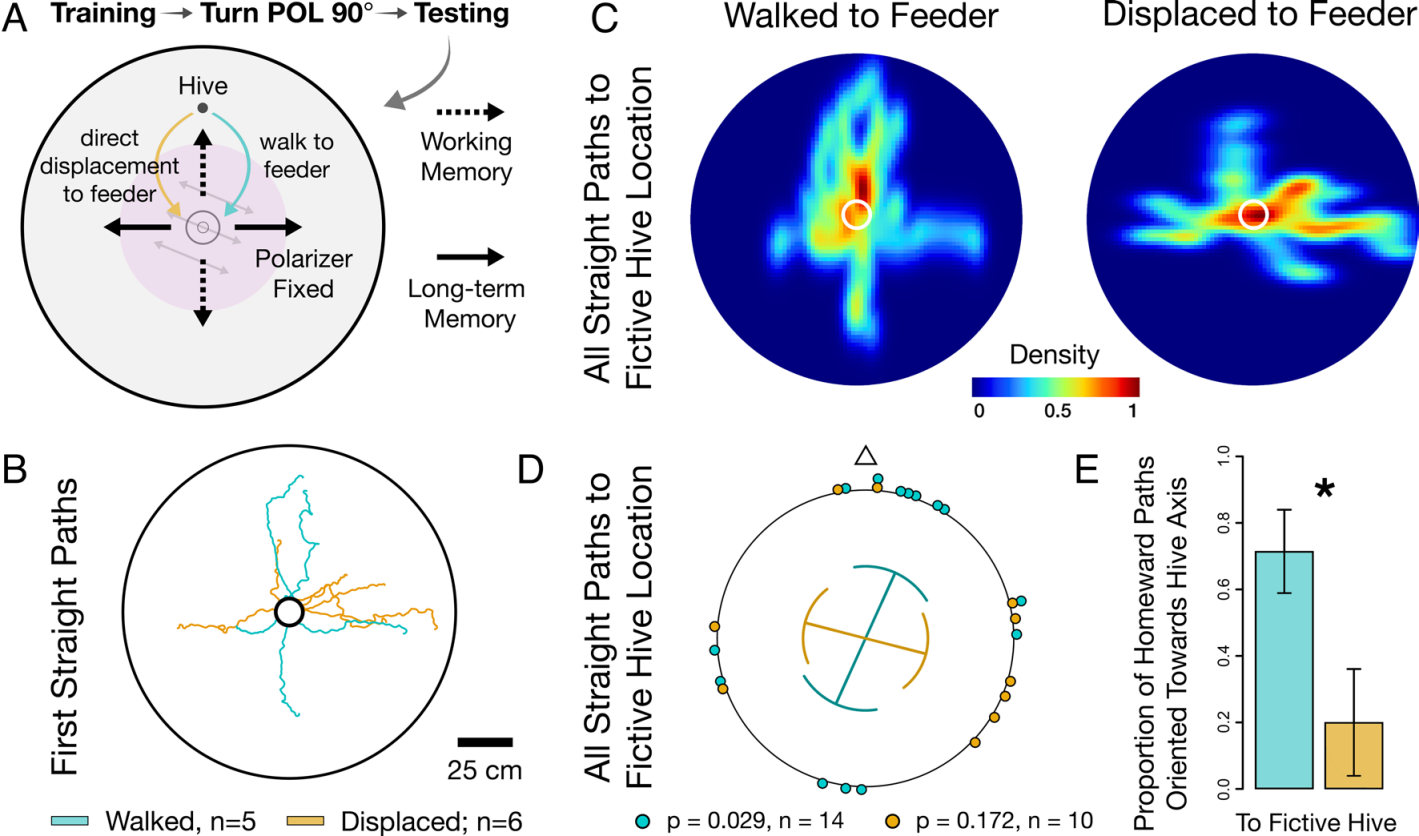
Path integration working memory is not required for homing in a featureless environment. (*A*) Following 3 d of training with a polarization field offset 90° from its testing configuration, bees either walked from the hive to the feeder or they were displaced to the feeder (eliminating the outward path). Bees were then trapped, displaced to a second identical arena, and allowed to navigate to the fictive hive position. Hive-oriented paths were predicted only for bees possessing a path integration vector in working memory, i.e., bees with an outbound path. Paths perpendicular to the hive direction were predicted if bees relied on information learned during the training period. (*B*) Path tracings of the first straight-line homeward path each bee enacted. (*C*) Heatmaps of all home vectors constrained up to the point that bees located the fictive hive position. Warmer colors: heavily trafficked areas. White circles: boundaries of feeder. (*D*) Orientations of all homeward paths constrained up to the fictive hive position (doubled data; walked to feeder: 48.70 ± 85.02°, *P* = 0.029; displaced to feeder: 208.97 ± 75.40°, *P* = 0.172). Lines within plot: circular means; curved sectors: circular SD; line lengths: strength of orientation (R-bar). (*E*) Mean proportion of homeward paths in *D* oriented in the axis of the hive for both experimental groups. Error bars: SEM. Asterisk (*): significant difference between groups (*P* = 0.020).

To clarify what information the bees were using to orient their homeward trips, we modified the above experiment. During the testing day, when the polarizer was oriented 90° from training days, bees were either allowed to walk to the feeder or were displaced directly from the hive entrance to the feeder so that they lacked an outward path. Without an outbound path, bees could not accumulate a path integration vector in working memory. Once at the feeder, bees were trapped and displaced to the center of a second, identical arena that lacked a hive entrance, where they were then allowed to navigate freely until they encountered the position where the hive would have been located if they had been in the first arena. Straight paths from the feeder would signal that the bees were following vector memories to the hive. Importantly, paths should only have been correctly oriented toward the hive if a path integration vector was present in working memory, i.e., only during trials when bees were allowed to walk to the feeder (Fig. 2*A*).

Bees in both experimental conditions performed straight-line paths, suggesting that vector information was present irrespective of the existence of a path integration vector in working memory. We found that bees performed more homeward paths oriented in the hive–feeder axis when they were allowed to walk to the feeder compared to bees that were deprived of outward paths to the feeder. Displaced bees were instead oriented primarily in the axis perpendicular to the hive direction (Fig. 2 *B–E* and *SI Appendix*, Tables S1 and S2). Thus, eliminating path integration working memory revealed the existence of an underlying long-term vector memory that enabled correct homing in relation to the polarization stimulus experienced during the preceding training days.

### Bumblebees Navigate Using Path Integration Vectors Stored in Long-Term Memory.

When examining the results between the first two experiments in detail, one notices an apparent contradiction. In the first experiment, bees that had experienced the unfamiliar, rotated polarizer during outbound journeys oriented preferentially 90° away from the hive, i.e., according to a long-term vector memory (black points Fig. 1*D*). Yet in the equivalent situation in experiment two (bees that had walked to the feeder), bees preferentially oriented to the actual hive position, i.e., using their path integration vector in working memory (cyan points Fig. 2 *C* and *E*). Besides being displaced to the second arena in experiment two, no differences between experimental methodology existed. However, we observed that the foraging intensity of the hive used for experiment two was generally much lower than the hive used for the first experiment, implying that bees from the second hive on average had less foraging experience during the training period. This suggests that the strength of long-term vector memories is variable and depends on the individual foraging experience of each bee. Hence, the performance of bees during test trials can be expected to be driven by a balance between working memory and long-term memory, with differences in experience shifting the bees to use one over the other.

**Fig. 3.**
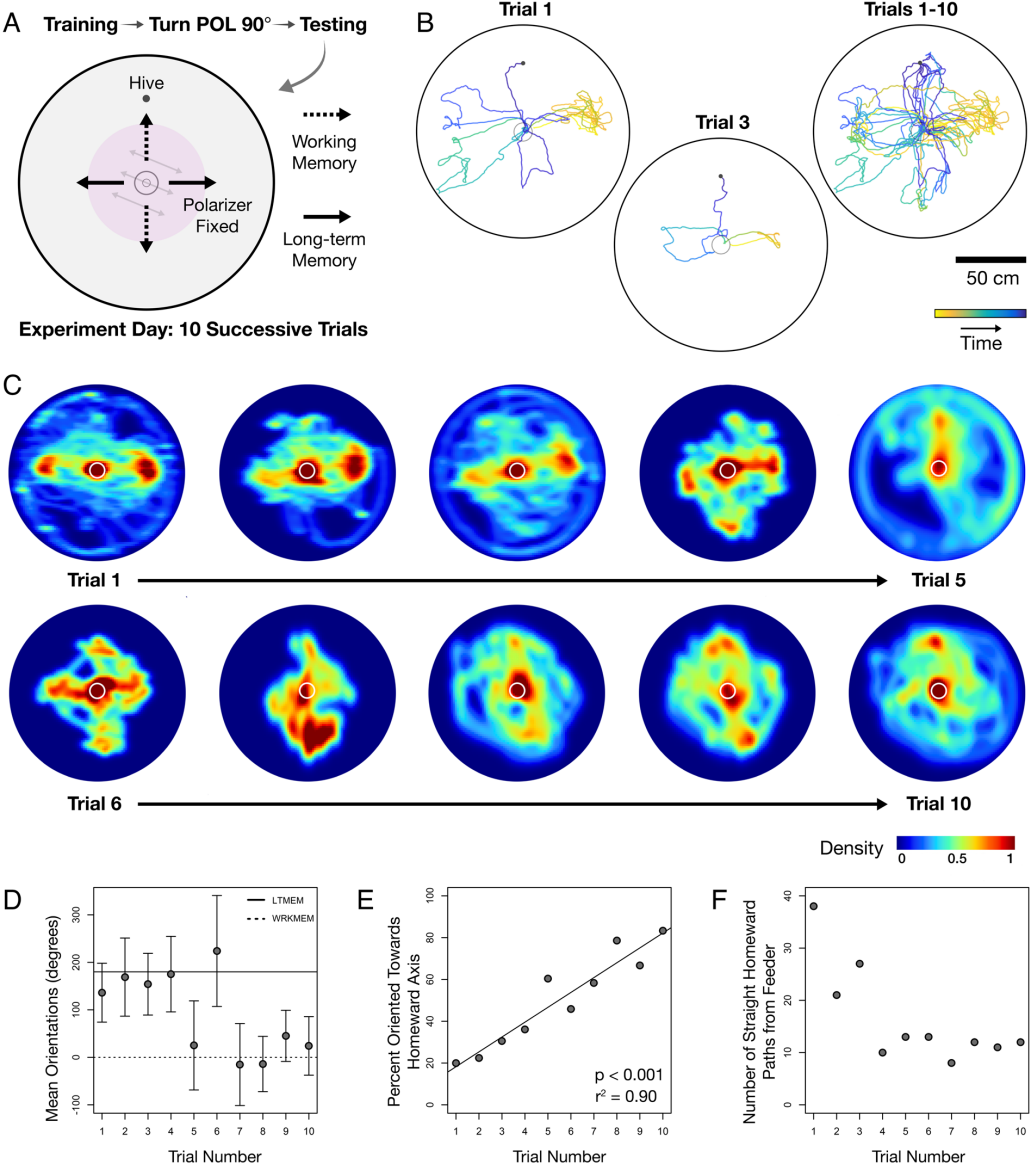
Bumblebees navigate with home vectors recalled from long-term memory. (*A*) An overhead polarized light field was offset 90° between the three-day training phase and the testing day. During testing, bees were allowed to enter the arena and locate the central feeder 10 consecutive times over a single day. Homeward paths were predicted to be oriented perpendicular to the direction of the hive if bees were homing using vectors stored in long-term memory. Alternately, home vectors oriented toward the hive should be products of path integration working memory. (*B*) Homeward path tracings of a single individual from the first and third time it entered the arena on the testing day. Compiled homeward path tracings from 10 consecutive trials this individual performed. (*C*) Search densities of all individuals compiled for each consecutive trial; Warmer colors: heavily trafficked areas. White circles: boundaries of feeder. (*D*) Circular means of the orientations of all home vectors traveled by each bee per trial (doubled). Error bars: SD. (*E*) Percentage of all homeward paths enacted by all individuals oriented toward the correct homeward axis per trial. (*F*) Numbers of homeward paths in *D* and *E* per trial across all six individuals.

We therefore next tested the relative impact of working memory vectors versus long-term memory vectors in bees of a single hive with high foraging intensity. As before, a polarized light field was fixed in place during 3 d of training and was positioned 90° from the training orientation during testing. Bees were then allowed to locate the central feeder and return home without manipulation. We tested each bee for 10 consecutive trials over a single day. Within each trial, bees were able to make several homing attempts until they successfully located the hive entrance. Homeward paths should have been oriented perpendicular to the direction of the hive if bees were homing using vectors stored in long-term memory, whereas homing paths oriented toward the hive should have resulted from vectors accumulated in path integration working memory (Fig. 3*A*).

During each trial, each bee attempted multiple homing paths with defined, straight headings. These bees would leave and return to the feeder if they had not encountered the hive entrance, continuing to carry out homing runs until they found the hive. The headings of these homeward paths were oriented in both expected directions—the directional axis of the hive as well as 90° away from it (Fig. 3*B*). Interestingly, we found that searches in earlier trials were focused around hive locations related to the stimulus position during the training phase, i.e., according to vectors recalled from long-term memory. In contrast, search densities in later trials were centered around predicted locations of the actual hive, with intermediate trials splitting search densities at both locations (Fig. 3 *C* and *D*). Moreover, within each trial, the proportion of homeward paths oriented in the hive–feeder axis rather than the axis perpendicular to it increased in a linear rate with increasing experience of the testing condition (*P* < 0.001; Fig. 3*E* and *SI Appendix*, Table S2).

These results support the hypothesis that bees were navigating home with vectors recalled from long-term memory that had been constructed during the training period. During early trials, after long-term memory vectors proved incorrect, bees would switch to their only other source of directional information, path integration vectors constructed in working memory in relation to the configuration of the polarization stimulus experienced during their outbound trip. With increased experience of the experimental condition, bees would more readily switch away from orienting according to their long-term vector memory constructed during training, instead favoring orienting toward the actual hive–feeder axis. There are two possible explanations for this transition: First, bees might have learned to disregard erroneous long-term vector memories and instead relied on path integration vectors in working memory. Second, they might have transferred successful working memory vectors into a new long-term memory during the first trials of the testing day. Over the course of the day, they would then increasingly rely on these newly memorized vectors over vector memories from the training phase. We next explored these two alternatives.

### New Parallel Vector Memories Are Formed Once Original Memories Become Ineffective.

**Fig. 4.**
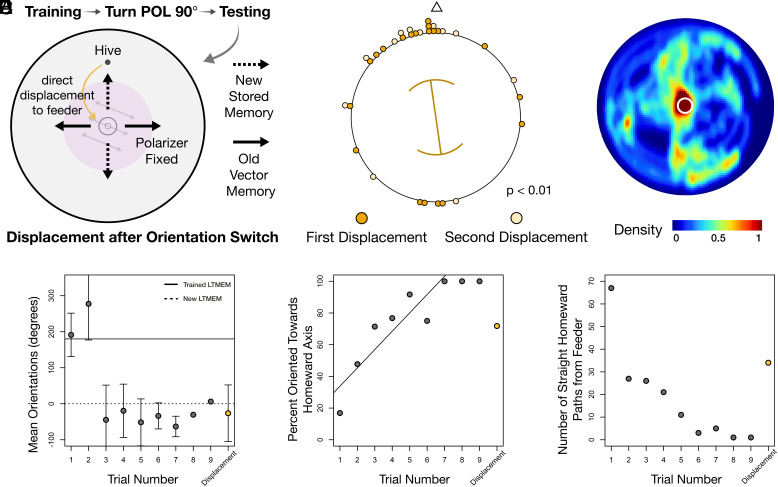
New parallel vector memories are formed once original memories become ineffective. (*A*) Experimental design as in Fig. 3 except that after two consecutive trials where bees oriented correctly toward the hive, bees were displaced from the hive entrance to the feeder to eliminate outward paths. During this trial, paths were predicted to be oriented toward the hive only if a new learned vector was present in long-term memory, constructed after the original long-term memory had become ineffective. (*B*) Orientations of homeward paths for displacement trials (doubled data; displacement 1: 339.77 ± 73.51°, *P* = 0.069; displacement 2: 340.75 ± 72.54°, *P* = 0.020; displacements pooled: 340.12 ± 72.77°, *P* = 0.0018). Lines within plot: circular means; curved sectors: circular SD; line lengths: strength of orientation (R-bar). (*C*) Search densities of all individuals for displacement trials; Warmer colors: heavily trafficked areas. White circle: boundary of feeder. (*D*) Circular means of orientations of all home vectors traveled by each bee per trial (doubled). Displacement trials are grouped together rather than by trial number. Displacements occurred between trials 4 to 10, mean: trial 6. Error bars: SD. (*E*) Percentage of all homeward paths enacted by all individuals oriented toward the correct homeward axis per trial. (*F*) Numbers of homeward paths in (*D* and *E*) per trial over 13 individuals.

To disentangle our two hypotheses for how bees were transitioning from orienting according to long-term memory vectors constructed during training to orienting in the actual hive–feeder axis, we further modified our experiment. In this new iteration, once bees oriented correctly in the axis of the hive for two consecutive trials, during the next two trials they were displaced from the hive entrance to the feeder so that they lacked an outward path and thus also lacked a working memory home vector. If bees during their last successful trial had learned to disregard their original long-term memories and instead relied on working memory to home, paths after the displacement should be either oriented perpendicular to the hive–feeder axis (defaulting to the original long-term memories constructed during training) or unoriented. In contrast, we predicted that homeward paths after displacements could only be oriented toward the actual hive if bees had stored successful home vectors constructed during the initial trials of the testing day in a new long-term memory, which they then preferentially used for homeward navigation (Fig. 4*A*).

We found that during displacement trials following the switch in orientation, bees showed straight-line paths that were primarily oriented in the direction of the hive (Fig. 4*B* and *SI Appendix*, Tables S1 and S3). This shows that bees did not rely on vectors from working memory to home toward the hive, but rather formed new long-term memories. Even though search densities were primarily focused around predicted locations of the actual hive, hive locations predicted by old long-term vector memories were still searched to a lesser degree as well, demonstrating that these memories were still intact. Thus, our results suggest that both long-term vector memories exist in parallel in the bees’ brains. We therefore conclude that bees that have walked to the feeder can actively choose to use one of three vector memories for steering along their homeward path: a path integration vector in working memory, the original long-term vector memory from the training phase, and the new long-term memory from the initial trials of the testing day.

Across all experiments it became apparent that these different vector memories competed with one another in complex ways. Our observations suggested that the extent of foraging experience determined the strength of the long-term memory and thus the extent to which it was used over working memory. For example, bees that showed high foraging intensity during training (Fig. 3 *D* and *E*) would rely on long-term memory vectors constructed during that period for more successive trials than bees which had been less motivated (Fig. 4 *D* and *E* and *SI Appendix*, Table S4). Additionally, reliance on erroneous long-term vector memories decreased linearly with increased experience to the testing condition (Figs. 3*E* and 4*E*). Together, these observations suggest that extended foraging experience results in an increased reliance on an associated vector memory.

## Discussion

We demonstrate that an insect can store multiple path integration–derived home vectors in long-term memory and can recall them at a familiar location to guide navigation. While we found bumblebees to be primarily reliant on vectors stored in long-term memory for homeward travel from a familiar feeder, working memory-derived path integration vectors were continuously present throughout foraging trips and were used when preferred long-term vectors proved unsuccessful. We showed that these working memory vectors can be transferred to long-term memory in parallel with existing long-term vectors, a process triggered by the failing of previously stored long-term vectors. The choice of information selected by the bee to guide homing behavior appears to be flexibly controlled by balancing homing success with the strength of the long-term memory.

Even though vector navigation is widespread among animals, the long-term vector memories our bumblebees exhibited are largely qualitatively different from vector memories described before in other insect species. For instance, in desert ants, stored path integration vectors are not retrieved at a familiar feeder (36). Though interestingly, our findings corroborate one result in honeybees in which vanishing bearings from a feeder were primarily oriented toward the trained direction of the hive rather than the direction of the hive indicated by the path integrator or a prominent landmark close to the feeder (37). Consistent with a fundamental difference in the memory type used to store vectors between bumblebees and other insects, vector memories in bumblebees reported earlier (9) were extremely stable, being maintained after chill-coma anesthesia. In contrast, induced chill-comas in desert ants (38) and dung beetles (39) degraded the behavioral output of path integration working memory (34, 35). However, differences in ecological adaptations between species that occupy deserts and higher latitudes may also explain differences in chill-coma resilience of vector memories.

Distinct from home vectors, navigational vectors over longer periods of time have been described in bees and ants, mostly to encode paths leading to feeders from the hive (1, 32–34). It is unclear how such food vectors are stored in memory, although evidence from honeybees (19, 30, 40–42) suggests either long-term vector memories or multiple working memories may be used in appropriate contexts. However, memorized views of the scenery along the foraging route (21, 31) may explain directional choices in some cases (19, 30). In desert ants, food vectors can be recalibrated when the feeder position is adjusted between foraging runs, resulting in intermediate orientations and suggestive of long-term storage (34, 43, 44). Additionally, ants lacking path integration working memory have been shown to recall site-specific traveling directions anchored to the global compass (local vectors), which are postulated to guide the ant to the next appropriate view during visual guidance (45). Similarly, honeybees flying over short distances have been shown to link learned segments of a path in a sequence along a route to a reward (46). In all cases, these data are distinct from our results: We excluded vectors linked to view matching strategies by eliminating asymmetrical visual features, vectors were always directed homeward from a feeder, and we never observed intermediate vectors when vector memories were conflicting. Instead, it appeared that distinct home vectors were assigned to separate long-term memories and it was the differential reliance on these vectors that was recalibrated. Importantly, these differences in the properties of vector memory match two core predictions for memories suited to construct complex vector maps (35). First, path integration-derived home vectors must be stored in long-term memory. Second, these home vectors need to be recalled at familiar locations. Both assumptions are met by the vector memories described by our results.

To build vector-based maps with more information than the path between a single feeder and hive, specific vectors would need to be associated with different locations. These independent vectors would then have to be recalled flexibly to allow effective navigation between any notable location and the nest. To enable novel shortcuts and to construct optimal traplining routes between multiple locations, associated long-term vectors would need to be compared through vector addition in the bee’s brain, particularly if those locations are sufficiently far from one another to lack shared visual features. Though alternately, an animal with the ability to store food vectors in long-term memory has been modeled to perform novel shortcuts using solely path integration without comparing multiple long-term vector memories, but instead adding a single food vector to the state of the path integrator when it wishes to travel to the associated food location (25). Novel shortcuts between locations emerge from this process. Preliminary behavioral evidence in desert ants supports these modeled results (47). However, this situation is less robust than one in which vector memories are recalled at familiar locations and interact, as a lost animal cannot recenter its vector reference until returning home. A vector map does not suffer such a limitation, allowing for a much more reliable and flexible navigation system. While testing those propositions is beyond the scope of the current study, our results demonstrate that bumblebees possess the basic elements of these complex abilities.

Independent evidence for the plausibility of maps based on vector operations results from studying the neural substrate of arthropod navigation, the central complex (CX). Connectomics data have revealed an ordered neuroarchitecture of this brain region which is predictive of computational algorithms implemented there (48). Consistent with physiological and anatomical data from bees (28), a main function of CX circuits is the comparison of current and desired headings of an animal to guide steering commands. Both directions appear to be represented as vectors, which are encoded as sinusoidal patterns of activity distributed across populations of CX neurons (49). Vector addition computations have been shown to occur in the CX to achieve spatial representations (50, 51), making the computations required for a vector map highly plausible. This principle was used in a neuroanatomically grounded model for path integration in the bee brain (28). Incorporating a single class of neurons designed for long-term storage of navigational vectors after associating them with learned valences transformed the simple path integration circuit model to one that encodes multiple vectors and uses them for vector computations (29). Interestingly, neurons anatomically matching these predictions exist in bees (52). As 15 to 20 of these cells were identified per brain hemisphere in the bumblebee, this provides an upper limit prediction of how many vectors a bumblebee would be able to store if these cells are indeed involved in vector retention. Our behavioral evidence of at least two parallel long-term vector memories in bumblebees, combined with identified neural specializations that match predictions from models reliant on vector computations, offers a clear path toward unraveling the neural basis of putative vector maps in insects.

If the long-term vector memories we revealed in bees are indeed used to construct vector-based maps, insects would be able to achieve high-level navigation behaviors without the need for the complex machinery underlying cognitive maps described in mammals. Rather, a neural circuit of only several hundred neurons can implement the comparably simple strategy of a vector map and thus overcome the restrictions imposed by the limited number of neurons found in insect brains without sacrificing much behavioral complexity, as theoretically shown in simulated agents (29). Given this efficiency, these insect neural circuits have the potential to inspire technological applications to solve complex navigational tasks with computationally and energetically inexpensive solutions, especially when combined with next-generation computing technology (53).

## Materials and Methods

### Experimental Subject Details.

Commercial hives of *Bombus terrestris* (Naturpol Smart, Koppert Biological Systems) were shipped to Lund University and were housed at room temperature under a 12:12 light:dark cycle. One day after arrival, the wings of all individuals were clipped. One day following clipping, the hive was introduced into the experimental arena and bees were allowed to freely forage. They were allowed access to a feeder with 1 M sugar-water solution and ground pollen (Bipollen EKO, Rawpowder Sweden AB) placed at the center of the arena. Foraging bees were labeled with unique small colored tags adhered to the back of the thorax. Data were collected from 82 individual bees from six hives, with each individual only being used once per experiment.

### Experimental Arenas.

Two relatively featureless, 1.5 m-diameter circular navigation arenas were constructed with a white composite wood base and were elevated 1.2 m to allow access below the arena (*SI Appendix*, Fig. S1). The first arena contained a hive entrance 25 cm from the arena’s periphery. The hive was placed beneath the arena and was connected to the arena by a tube with three doors to have control of bees that wished to enter the arena. Sugar water and pollen were placed in a removable conical feeder at the center of the arena. The conical feeder had a door which could slide over the bee while it was feeding, trapping it in the feeder. Both the feeder and the hive entrance were placed below ground-level of the arena so they were not visually detectable by bumblebees while in the arena. Trials were recorded from above using a raspberry pi camera connected to a raspberry pi unit (Raspberry Pi Foundation) outside of the arena, where experiments were observed and trials recorded.

The second arena was identical to the first with the exception that no hive or hive entrance was present and five potential locations were present where a conical feeder could be placed (one at the center and four near the edge of the arena). When a feeder was not occupying any of these potential feeder locations, a tight plug, level with the arena floor and constructed of the same material as the base of the arena was present instead.

Arenas were placed in a dark room and surrounded by thick matte black curtains and lit from above using a centered, diffused, custom-built light emitting diode (LED) light source with sets of ultraviolet (40 units, LZ1-10UV0R, LED Engin Inc.) and white LEDs (37 units, LZ1-10CW02, LED Engin Inc.). Composite filters constructed of a linear polarizer (38% transmission neutral gray, Rosco Laboratories Inc.) and two sheets of tracing paper were placed under each light source. When the polarizer side of the composite sheet faced downward toward the arena, light was linearly polarized to an average degree of 99.78% from 350 to 600 nm (98.23% from 350 to 450 nm; *SI Appendix*, Fig. S1*D*). The overhead polarization stimulus had an angular diameter of 22.6° when viewed at the ground level from the center of the arena and could be rotated up to 90° from outside of the arena. Irradiance spectra of all light sources can be reviewed in *SI Appendix*, Fig. S1*C* (from ref. 9). Arenas from Patel et al. (9) were used in the current study.

### Experimental Procedures.

Hives containing bees with clipped wings were introduced into the experimental arena and bees were allowed to freely forage for 3 d with a linearly polarized light field present overhead and a feeder with 1 M sugar water and pollen at the center of the arena. During the first day, each individual bee that emerged from the hive was labeled with a colored tag. After the free-foraging period, the doors to the hive were closed and experiments were conducted.

During the polarization orientation experiments, an overhead linearly polarized light field was fixed in place during 3 d of training. During testing, the polarized field was either initially positioned as during training or 90° from this orientation. Bees were then let out of the hive individually, allowed to locate the feeder, and were trapped in the feeder. Once bees were trapped, the polarizer was rotated 90° from its original position and the bee was released. In control trials, the polarizer was rotated 45° forward and backward by the researcher, so it ended at its original position (Fig. 1*B*). Experimental conditions were performed in a randomized order.

To determine whether bees required path integration working memory to navigate to the hive, the overhead polarized light field was fixed in place during 3 d of training. Before testing, the polarizer was positioned 90° from the training orientation. During testing, bees were either allowed to walk to the feeder or they were displaced from the hive entrance to the feeder so that they had lacked an outward path. Once at the feeder, bees were trapped and displaced to a second identical arena and were allowed 10 min to navigate in the second arena (Fig. 2*A*). Homeward paths were then constrained up to the point where bees reached the fictive hive position in the arena to be comparable to experiments where the hive entrance was present. Experimental conditions were performed in a randomized order.

To examine the relative impact of working memory vectors versus long-term memory vectors, the overhead polarized light field was offset 90° between training and testing periods. During testing, bees were allowed to enter the arena and locate the central feeder and return to the hive without manipulation. Bees were allowed to enter the arena 10 consecutive times over a single day (Fig. 3*A*). One bee only completed two trials and was thus excluded from analyses (*SI Appendix*, Table S4).

To test hypotheses investigating what sources of information bees were increasingly relying on with increased experience during the previous experiment, the exact same experimental design as in the previous experiment was replicated with one key exception: During the two trials following two consecutive trials where bees oriented correctly toward the hive from the feeder, bees were displaced from the hive entrance to the feeder so that they had lacked an outward path (Fig. 4*A*). Thus, only bees that completed the procedure up to trials where they were displaced were included in analyses (*SI Appendix*, Table S4).

### Quantification and Statistical Analysis.

As done by Patel et al. (9), foraging paths between the hive and the feeder were video recorded at 10 frames per second. Videos were unwarped to correct for lens distortion and distortion due to camera placement using the Camera Calibration Toolbox in Matlab (vR2020a, MathWorks Inc.). To differentiate homeward paths from continued arena exploration, paths from the feeder were considered to be homeward paths when a straight path started at most 14 cm from the feeder and when they did not deviate more than 90° from their initial trajectories for at least 28 cm. From these homeward paths, search behaviors were determined to be initiated when an animal turned more than 90° from its initial trajectory and continued walking at a distance of at least 14 cm. The number of trials with homeward paths per experimental condition is reported in *SI Appendix*, Table S4.

Paths were automatically tracked using the software ivTrace (v2.23, Bielefeld University), from which the output is given as Cartesian coordinates. From these measures, search densities were visualized using R (v4.0.2, R Core Development Team) with the “autoimage,” “ggforce,” “ggplot2,” and “gridExtra” plugins.

Additionally, the orientation of homeward paths when bees were 28 cm from the feeder in relation to the hive entrance was recorded using the angle tool in ImageJ (v2.3.0, Fiji).

All statistical analyses were run on R (v4.0.2, R Core Development Team) with the autoimage, “car,” “circular,” “CircMLE,” ggplot2, “lme4,” “plotrix,” and “shape” plugins. All experiments were conducted under a polarized light field, resulting in expected bimodal orientations. Thus, all statistical analyses for experiments were performed after using the doubling angles procedure for bimodal data as outlined in Batschelet (54). Effectively, this transformation converts axial data of 180° periodicity into circular data with 360° periodicity, so that standard circular statistics apply. Maximum likelihood estimates of model fitting for modal models of data structure (55) were used to justify the pooling of data presented in Fig. 4*B* (*SI Appendix*, Table S3). Orientation data were analyzed using the following procedures for circular statistics (54): Rayleigh tests of uniformity were used to determine whether homeward paths were oriented within a group for all experiments. Watson two-sample tests for homogeneity were used to determine whether group orientations were significantly different from one another. All reported mean values for orientation data are circular means and circular SD (*SI Appendix*, Table S1).

Generalized linear mixed modeling was used to analyze the data presented in Figs. 2*E* and 3*E*. Our first model used the axial orientation of homeward paths during testing as the variable of interest and experimental condition (walking or displacement to feeder) as the predictor variable, specifying a binomial error distribution (Fig. 2*E*). Our second model used the axial orientation of homeward paths during testing as the variable of interest and trial number as the predictor variable, specifying a binomial error distribution as well (Fig. 3*E*). Since individual bumblebees made multiple homeward paths from the feeder, all models included the identity of the individual performing each homeward path as a random term even though it did not significantly increase the explanatory power of any of our models (*SI Appendix*, Table S2).

Bonferroni corrections were used when applicable. All statistical information including sample sizes, test statistics, *P*-values, means, SD, and outcomes of linear models are presented in *SI Appendix*, Tables S1–S3.

## Supplementary Material

Appendix 01 (PDF)

## Data Availability

The data from this manuscript are published as an experiment entry in the InsectBrainDatabase (56) under identification numbers EIN-0000273, EIN-0000274, EIN-0000275, and EIN-0000276.
