# Supplementary material for: Parallel vector memories in the brain of a bee as foundation for flexible navigation
Source: Proc Natl Acad Sci U S A. 2024 Jul 15;121(30):e2402509121. doi: 10.1073/pnas.2402509121 (PMC11287249; doi:10.1073/pnas.2402509121)
Supplement: Supplementary file 1 — Appendix 01 (PDF) [file pnas.2402509121.sapp.pdf]

Supporting Information for

Parallel vector memories in the brain of a bee as foundation for flexible navigation

Rickesh N. Patel[1]*, Natalie S. Roberts[1], Julian Kempenaers[1], Ana Zadel[1], Stanley Heinze[1,2]

1 Lund Vision Group, Department of Biology, Lund University; Lund, 22362, Sweden.

2 Nano Lund, Lund University; Lund, 22362, Sweden.

*Corresponding author: Rickesh N. Patel

Email: rickesh.patel@biol.lu.se*

This PDF file includes:

Figures S1 to S2

Tables S1 to S4

SI References

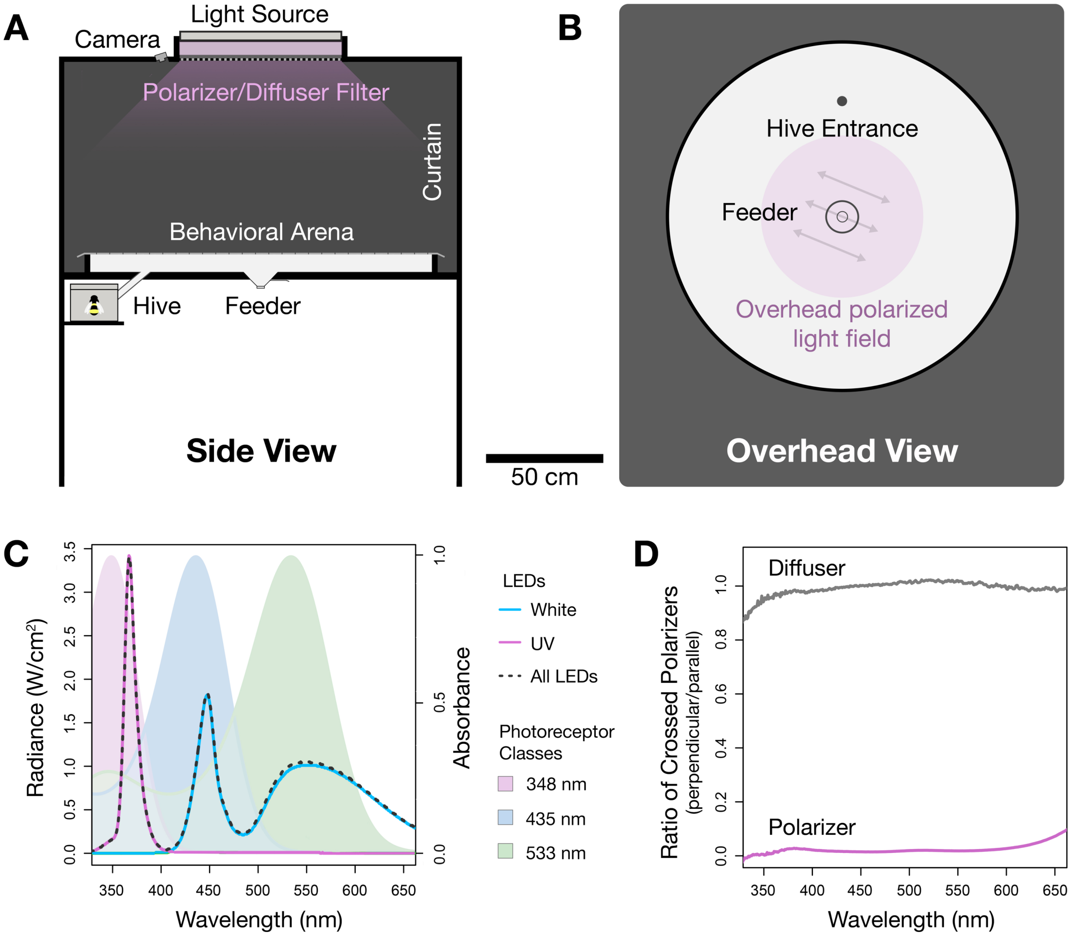

Fig. S1. Navigation arena design. (A) Side view. **(B)** Overhead view. The circular arena (150 cm diameter) was directly connected to a hive via an entrance located 25 cm from the arena's periphery (filled circle in B). Sugar water and pollen were provided in a conical feeder at the arena center. Neither the feeder nor hive-entrance were visible to bees in the arena. The arena was illuminated by an overhead light source that could either be highly polarized or depolarized. Experiments were video recorded from above. **(C)** Irradiance spectra of light available at the center of the arena (dashed line). Violet and blue curves: irradiance of the isolated UV and white LEDs from the overhead light source. Curves with colored area under the curve: Absorbance spectra of photoreceptor classes in the eyes of *Bombus terrestris dalmatinus* (from Skorupski et al. (54)). **(D)** Ratio of light transmitted through the overhead filter and a second crossed polarizer (perpendicular orientation/parallel orientation) when either the polarizer side (violet) or diffuser side (grey) faced towards the arena.

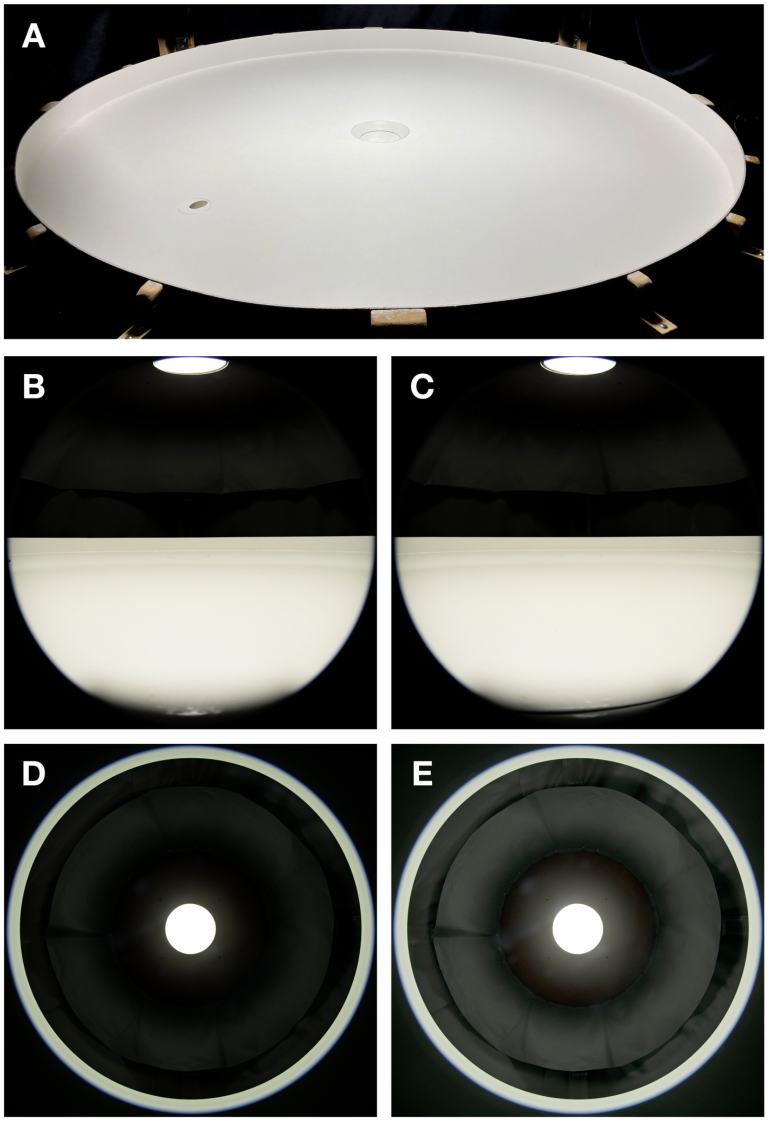

Fig. S2. Photos of the navigation arena. Illuminated by **(A)** the overhead UV and white LEDs transmitted through the polarized filter. **(B)** Full hemispherical photo of the behavioral arena taken from the center of the arena oriented towards the hive at horizon with a fisheye lens. **(C)** Same photo as in (B) oriented 90° from the hive at horizon. **(D)** Full hemispherical photo of the behavioral arena taken from the center of the arena oriented towards zenith with a fisheye lens. **(E)** Same photo as in (D) overexposed.

Table S1. Summary of orientation statistics for all experimental groups. Related to Figures 1CD, 2D, and 4B. Orientations were analyzed using Rayleigh Tests of Uniformity. Significance indicates that groups are oriented in a single direction. Only orientations of straight-line paths (see Methods) were used for analyses. Analyzed using doubled data. *When two groups were significantly oriented, their relative orientations were compared using a Watson Two-Sample Test of Homogeneity (last row). Significance indicates that groups are oriented differently from one another.

Experiment	P-value	N	R-bar	Mean ± SD
**Orientation to polarized light (Figure 1)**				
Polarized Field Fixed (polarizer same as training)	<0.001	12	0.813	353.26° ± 36.9°
Polarized Field Rotated 90° (polarizer same as training)	<0.001	13	0.850	161.75° ± 32.6°
Polarized Field Fixed (polarizer 90° from training)	0.069	17	0.395	150.115°±78.09°
Polarized Field Rotated 90° (polarizer 90° from training)	0.135	14	0.379	349.29°±79.87°
**Long-term vector memory: displacement during trial 1 (Figure 2)**				
Walked to hive (to fictive hive)	0.029	14	0.496	48.70° ± 85.02°
Displaced to hive (to fictive hive)	0.172	10	0.421	208.97° ± 75.40°
**Long-term vector memory: displacement after axis switch (Figure 4)**				
Displacement 1	0.069	20	0.364	339.77° ± 73.51°
Displacement 2	0.020	12	0.559	340.75° ± 72.54°
Displacements Pooled	0.0018	32	0.437	340.12° ± 72.77°
**Watson Two-Sample Test of Homogeneity (Figure 1)**			**P-value**	**s**
Polarized Field: Fixed vs. Rotated 90° (polarizer same as training)*			<0.001	0.527

Table S2. Summary of the generalized linear mixed models used in Figures 2E and 3E. P < 0.05 indicate a significant effect of the predictor variable on the variable of interest. Incorporating the identity of the individual performing each homeward path did not significantly increase the explanatory power of any models (results of ANOVA analyses between models).

Model	P-value	Test Statistic	Coefficient Estimate
**Displacement Trial 1: to fictive hive (Model 1, related to Figure 2E)**<br>Variable of Interest: Axial orientation of homeward path<br>Predictor Variable: Walked or displaced to feeder<br>Random Term: Individual<br>Error Distribution: binomial	0.0197	Z = 2.332	0.987
**Displacement Trial 1: to fictive hive (Model 2, related to Figure 2E)**<br>Variable of Interest: Axial orientation of homeward path<br>Predictor Variable: Walked or displaced to feeder<br>Error Distribution: binomial	0.0197	Z = 2.332	0.987
**ANOVA of Model 1 and Model 2**	1	$c^2 = 0$	DAIC = 2
**Axial Orientation per Trial (Model 1, related to Figure 3E)**<br>Variable of Interest: Axial orientation of homeward path<br>Predictor Variable: Trial number<br>Random Term: Individual<br>Error Distribution: binomial	<0.001	Z = 4.938	0.205
**Axial Orientation per Trial (Model 2, related to Figure 3E)**<br>Variable of Interest: Axial orientation of homeward path<br>Predictor Variable: Trial number<br>Error Distribution: binomial	<0.001	Z = 5.214	0.198
**ANOVA of Model 1 and Model 2**	0.253	$c^2 = 1.309$	DAIC = 0.7

Table S3. Summary of the maximum likelihood estimates of modal circular data structure for data used in Figure 4B. The top best fit distribution models for both isolated displacements and displacements pooled together are the same. Therefore, an argument can be made for pooling both displacements.

**First Displacement**	**AIC**	**DAIC**
M2B: Symmetric modified unimodal	61.37	0
M3B: Symmetric bimodal	63.22	1.85
M2C: Modified unimodal	65.22	3.85
M4B: Axial bimodal	65.93	4.56
M4A: Homogenous axial bimodal	67.81	6.44
M5B: Bimodal	68.78	7.41
M5A: Homogenous bimodal	69.38	8.01
M3A: Homogenous symmetric bimodal	69.98	8.60
M2A: Unimodal	72.63	11.26
M1: Uniform	73.52	12.14
**Second Displacement**	**AIC**	**DAIC**
M2B: Symmetric modified unimodal	40.73	0
M3B: Symmetric bimodal	42.72	2.00
M2C: Modified unimodal	43.00	2.27
M2A: Unimodal	43.58	2.85
M4A: Homogenous axial bimodal	44.11	3.38
M1: Uniform	44.69	3.96
M4B: Axial bimodal	44.84	4.11
M3A: Homogenous symmetric bimodal	45.31	4.58
M5B: Bimodal	45.54	4.81
M5A: Homogenous bimodal	46.69	5.96
**Displacements Pooled**	**AIC**	**DAIC**
M2B: Symmetric modified unimodal	99.87	0
M3B: Symmetric bimodal	101.78	1.91
M2C: Modified unimodal	101.84	1.97
M4B: Axial bimodal	103.78	3.91
M4A: Homogenous axial bimodal	104.00	4.13
M5A: Homogenous bimodal	106.00	6.13
M2A: Unimodal	108.38	8.51
M3A: Homogenous symmetric bimodal	108.89	9.01
M5B: Bimodal	111.35	11.47
M1: Uniform	117.62	17.75

Table S4. Number of individuals that first oriented in the axis predicted from a long-term vector memory and that completed enough trials to meet the requirement of the experiment for all long-term vector memory experiments. Related to Figures 2, 3, and 4. Note that hive five had very few individuals who oriented as predicted from a recalled long-term vector memory. Limited foraging trips during the training period may explain why some individuals initially oriented as predicted from a path integration vector constructed in working memory rather than a long-term vector memory.

**Hive: Experiment: Associated Figure**	**Number of individuals first oriented towards a long-term vector memory**	**Number of individuals that completed experiment**
2: Displacement Trial 1- Walked to feeder: Fig 2	2/5	5/5
2: Displacement Trial 1- Displaced to feeder: Fig 2	6/6	6/6
3: Long-term vs Working memory: Fig 3	7/7	6/7
4: Displacement after axial orientation switch: Fig 4	13/15	7/13
5: Displacement after axial orientation switch: Fig 4	3/13	3/3
6: Displacement after axial orientation switch: Fig 4	4/5	3/4

SI References

1. P. Skorupski, T. F. Döring, L. Chittka, Photoreceptor spectral sensitivity in island and mainland populations of the bumblebee, *Bombus terrestris*. *J Comp Physiol A Neuroethol Sens Neural Behav Physiol* **193**, 485–494 (2007).
